# Modeling Alzheimer’s disease in progeria mice. An age-related concept

**DOI:** 10.1080/20010001.2018.1524815

**Published:** 2018-10-04

**Authors:** Kavita Sharma, Martin Darvas, C. Dirk Keene, Laura J. Niedernhofer, Warren Ladiges

**Affiliations:** a Department of Comparative Medicine, School of Medicine, University of Washington, Seattle, WA, USA; b Department of Pathology, Division of Neuropathology, School of Medicine, University of Washington, Seattle, WA, USA; c Institute on the Biology of Aging and Metabolism, Department of Biochemistry, Molecular Biology and Biophysics, University of Minnesota, Minneapolis, MN, USA

**Keywords:** Alzheimer’s disease, aging, progeria, mouse models

## Abstract

The prevalence of Alzheimer’s disease (AD) is expected to dramatically increase in older people worldwide. Efforts to find disease-modifying treatments have been largely unsuccessful because of the focus on disease-specific pathogenesis, and lack of animal models to study AD in the context of aging and age-related co-morbidities. The geroscience approach to studying AD would suggest that modulation of aging *per se* would be a useful strategy, but a mammalian model system that combines both aging and AD is not available. One approach to study old age and AD is to utilize murine models of progeroid syndrome, which can provide a number of advantages not only for basic aging biology but also for preclinical drug testing. A progeria background, such as the *Ercc1* mutant mouse (*Ercc1^−/Δ^*), provides an aging component not seen in current murine models of AD that lack age-related co-morbidities typical of AD patients.

*Ercc1^−/Δ^* mice experience the same types of stochastic endogenous DNA damage as WT mice, but accumulate lesions faster due to impaired DNA repair, which accelerates the normal aging process by 6-fold. These mice do not show frank AD pathology but represent a predisposed or hypersensitive environment for AD pathology, where pathogenic elements of AD can be introduced, either by crossing with well-established AD transgenic mouse lines, or transcranial stereotaxic delivery directly into the brain. Since *Ercc1^−/Δ^* mice age five to six times faster than WT mice, very rapid characterization and testing of therapeutic interventions is possible. Studies are urgently needed to capitalize on the highly informative potential of this novel AD mouse model.

Advanced age is the number one risk factor for many neurodegenerative diseases including sporadic Alzheimer’s disease (AD). Aging is also the number one risk factor for the majority of other chronic diseases. Ninety percent of persons over 65 years of age have one or more chronic diseases. Even more remarkable is the fact that 70 per cent of persons over 65 will have two or more diseases diagnosed as cardiovascular disease, chronic kidney diseases, muscle wasting, peripheral neuropathy, cognitive decline or other age associated disorders (National Council on Aging). Hence AD does not often occur in individuals as a singular disease, but is more likely to present in an individual with multiple co-morbidities. The prevalence of AD is expected to soar with the dramatic rise in the number of elderly individuals in both developed and developing countries. Efforts to find disease-modifying treatments have been largely unsuccessful. This lack of success is largely attributable to: i) focusing primarily on disease-specific pathogenesis, and ii) lack of animal models to study AD in an aged organism or in the context of other age-related co-morbidities. The geroscience approach to studying age-related diseases such as AD would suggest that modulation of aging *per se* may be a useful strategy for delaying the onset or retarding the progression of disease [1–3]. This concept is supported by an impressive body of knowledge identifying genetic, dietary and pharmacologic interventions that profoundly retard aging and its pathophysiologic effects in a number of invertebrate and vertebrate model systems [4]. Correlative human data suggest that the results in model organisms are translatable. To study how aging affects AD, and then how therapeutically targeting aging affects AD, requires an experimental model system that combines both aging and AD.

One approach to study old age and AD is to utilize murine models of progeroid syndromes, which can provide a number of advantages not only for basic aging biology but also for preclinical drug testing (Figure 1). There are dozens of human progeroid syndromes that have been accurately modeled in the mouse. This includes Werner, Cockayne syndrome, Hutchinson-Gilford progeria syndrome and XFE progeroid syndrome [5,6]. *Ercc1* mutant mice (*Ercc1^−/Δ^*), which model XFE progeroid syndrome, accumulate oxidative DNA damage in their cells, and senescent cells in their tissues [7] more rapidly than wild-type (WT) mice. They also show increased abundance of reactive oxygen species compared to age-matched WT mice, analogous to the differences between old and young WT mice [7]. Thus, these diseases appear to reflect a true compression of the normal aging process [8]. Mouse models of these diseases therefore offer a unique and very rapid system for discovering how aging causes predisposition to diseases such as AD. In fact, murine progeria models may mimic human aging to a greater extent than aged wild-type (WT) mice [6,]. *Ercc1^−/Δ^* mice that model XFE progeroid syndrome develop conditions common in elderly humans such as osteoporosis, pulmonary fibrosis, chronic kidney disease, cardiovascular disease, muscle wasting, peripheral neuropathy, hepatic fibrosis, urinary incontinence, intervertebral disc degeneration, cognitive decline, and loss of hearing and vision [6,9–13]. In addition, multiple therapeutic interventions have been demonstrated to extend the health span of *Ercc1^−/Δ^* mice [14,15], including anti-geronic therapeutics and senolytics [16,17]. This establishes *Ercc1^−/Δ^* mice as a rapid model system for identifying therapeutics that delay age-related diseases.

**Figure 1.**
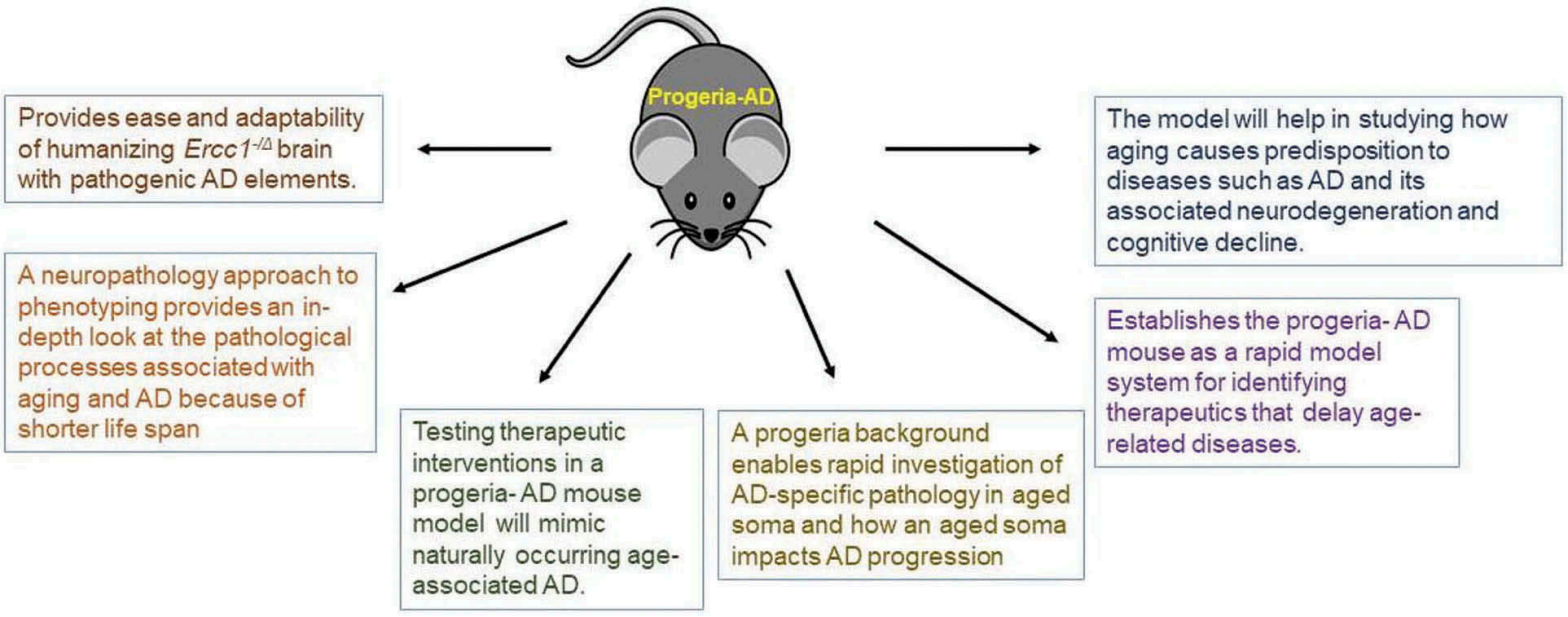
Advantages of studying Alzheimer’s disease in the *Ercc1*^−/Δ^ progeria mouse.

Accelerated aging in *Ercc1^−/Δ^* mice is presumed to result from failure to repair spontaneous oxidative DNA damage that occurs as a consequence of normal metabolic process. *Ercc1* encodes one subunit of a DNA repair endonuclease ERCC1-XPF, which is required for multiple DNA repair pathways including nucleotide excision repair [18], inter-strand crosslink repair [19], and double-strand break repair [20]. *Ercc1^−/Δ^* mice experience the same types of stochastic endogenous DNA damage as WT mice, but simply accumulate lesions faster due to impaired DNA repair [21]. This accelerates the normal aging process by 6-fold [8]. Importantly, the genotoxic stress driving accelerated aging in *Ercc1^−/Δ^* mice is physiologically relevant and occurs at physiological rates rather than being triggered by an acute, exogenous, supraphysiological exposure.

Increased oxidative DNA damage has been observed in subjects with mild cognitive impairments as well as late-Alzheimer’s Disease [22,23], suggesting a correlation between age-related accumulation of DNA damage and cognitive decline. Global *Ercc1* mutants as well as neuron-specific *Ercc1* mutants exhibit an age-dependent decrease in neuronal plasticity, and progressive neuronal pathology, suggestive of neurodegenerative processes [24]. These mice do not show frank AD pathology (Keene et. al, unpublished observations), but represent a predisposed or hypersensitive environment for AD pathology. The systemic aging of *Ercc1^−/Δ^* mice is ideal for creating a more clinically relevant murine model of AD if the pathological elements of AD are introduced.

There are several approaches for integrating pathologically relevant AD components into

*Ercc1^−/Δ^* mice to develop a progeria AD mouse model. Breeding schemes for *Ercc1^−/Δ^* mice have been established so it is relatively straightforward to cross with several existing transgenic mouse lines that have neuropathologic changes similar to AD (e.g. models of amyloid beta or tau pathology). One example would be crossing the APPswePS1Δe9 transgenic mouse line, a well characterized and highly used model for AD amyloid pathology, into a C57BL6 *Ercc1^−/Δ^* background. APPswePS1Δe9 transgenic mice would be ideal for crossings with *Ercc1^−/Δ^* mice, because they express mutant APP and PSEN1 forms that are inherited as one genetic block, hence providing a simple and efficient breeding scheme. Therefore, the APPswePS1x *Ercc1^−/Δ^* BL6 can be crossed with *Ercc1^−/Δ^* FVB mice to create APPswePS1x *Ercc1^−/Δ^* progeroid mice in an f1 background. This would provide the opportunity to study the effect of beta amyloid on triggering cognitive dysfunction and neuropathology in a background of accelerated aging, which could very well underlie an individual’s susceptibility to disease processes typical of AD. Another approach is a transcranial model of AD. We have successfully generated AD lesions in old mice based on adeno-associated virus (AAV)-mediated Aβ42 expression, introduced into the hippocampus by stereotactic injection using published procedures [25,26]. We have also performed stereotaxic injections into the brains of *Ercc1^−/Δ^* mice and are able to detect robust expression of Aβ42, gliosis, and astrocytosis in the injected and surrounding areas.

Relevant phenotypic assessment is necessary to validate any animal model of AD. Many AD mouse models currently in use and being published have not been properly phenotyped from a neuropathological perspective as, it relates to AD pathological distribution and progression, based on the NIA-AA consensus criteria for assessment of AD [27]. This impedes successful translation of pharmaceutical studies. A neuropathology approach to phenotyping provides an in-depth look at pathological processes, and standardized phenotyping yields procedures that are highly reproducible from lab to lab and translatable from mouse to human. We have adapted the NIA-AA neuropathology guidelines [28,29] to the mouse [27], and are applying them for sampling and staining techniques so that neuronal tissues are characterized according to the same standardized approach with maximal relevance to human disease. Behavioral testing procedures, which are available to measure cognitive function in the mouse [30,31], can be used to correlate learning and memory deficits with the development and progression of neuropathological lesions.

In addition to neuropathology, the effect of systemic age-related pathology on central nervous system function and the susceptibility to and progression of AD is of interest. We have extensive experience in the geropathological assessment of tissues from aged mice, as well as tissues from progeria mice [32–35]. The progeria in *Ercc1^−/Δ^* mice is systemic, affecting all organ systems including the CNS, enabling a broad examination of the effect of multiple age-related diseases and loss of tissue homeostasis on AD and its treatment. Age-related phenotypes begin in young adult *Ercc1^−/Δ^* mice and progress until death at 7 months of age [6]. The penetrance of the phenotype is 100% and highly predictable. Using the Geropathology Grading Platform [34], we have shown that *Ercc1^−/Δ^* mice have more severe lesions in multiple organs than age-matched WT controls, similar to old vs. young WT mice.

In summary, a progeria background provides an aging component not seen in current murine models of AD, since disease is generally triggered in young adult animals lacking age-related co-morbidities typical of AD patients. It also provides a system for rapid investigation of how AD-specific pathology impacts an aged soma and how an aged soma impacts AD progression. Studying AD in murine models of human progeria, specifically *Ercc1^−/Δ^* mice, offers the advantage that these mice age five to six times faster than WT mice, enabling rapid characterization and testing of therapeutic interventions. A progeria AD mouse model would therefore improve drug testing because it better models sporadic AD and can be completed in less than six months. Studies are urgently needed to capitalize on the highly informative potential of this novel AD mouse model.
